# Suicide Risk Among Patients With Cancer by Sex in Japan: A Population-based Study

**DOI:** 10.2188/jea.JE20230280

**Published:** 2024-11-05

**Authors:** Shinichi Kitagawa, Tomotaka Sobue, Ling Zha, Toshitaka Morishima, Yuko Ohno, Isao Miyashiro

**Affiliations:** 1 Environmental Medicine and Population Sciences, Department of Social Medicine, Graduate School of Medicine, Osaka University, Osaka, Japan; 2 Cancer Control Center, Osaka International Cancer Institute, Osaka, Japan; 3 Division of Health Science, Graduate School of Medicine, Osaka University, Osaka, Japan

**Keywords:** neoplasm, prognosis, self-injurious behavior, social problem, epidemiology

## Abstract

**Background:**

In Japan, few studies have examined suicide risk for 5-year relative survival rates for cancer sites. Since 5-year relative survival rates differ by sex, we aim to examine suicide risk for patients with cancer separately for men and women.

**Methods:**

We estimated the risk of suicide among patients with cancer by sex in Japan compared to the general population, using standardized mortality ratios (SMRs). Patients with cancer diagnosed between January 1, 1985–December 31, 2013 and registered in the Osaka Cancer Registry were followed for up to 10 years. The outcome was suicide death. In addition, cancer sites were classified into three prognosis groups based on 5-year relative survival rates: good (>70%), moderate (40–70%), poor (<40%).

**Results:**

Among 623,995 patients with cancer observed for 2,349,432 person-years, 1,210 patients died by suicide (867 men and 343 women). The SMRs were almost equal for men (1.66; 95% confidence interval [CI], 1.55–1.77) and women (1.65; 95% CI, 1.48–1.83). SMRs for cancer prognosis groups were 1.01 (95% CI, 0.84–1.22) for men and 1.47 (95% CI, 1.24–1.73) for women in the good group, 1.53 (95% CI, 1.39–1.68) for men and 1.74 (95% CI, 1.47–2.05) for women in the moderate group, and 2.54 (95% CI, 2.27–2.85) for men and 1.87 (95% CI, 1.43–2.46) for women in the poor group.

**Conclusion:**

In this population, both sexes had higher suicide risk with poor prognosis, but the difference in SMRs between the good and poor groups was smaller for women than men.

## INTRODUCTION

With more than 700,000 suicide deaths worldwide each year,^1^ suicide is a public health issue. Among the Organisation for Economic Co-operation and Development (OECD) countries, Asian countries, such as South Korea and Japan, have higher suicide rates than European and North American countries.^2^ In addition, the 1997 Asian currency crisis increased male suicide rates in South Korea, Hong Kong, and Japan in 1998.^3^^,^^4^ During the recent coronavirus disease 2019 (COVID-19) pandemic, suicide rates among Japanese men and women increased,^5^ indicating the need for ongoing suicide prevention efforts.

In contrast to the general population, patients with cancer have a higher risk of suicide.^6^^–^^19^ Heinrich et al, in a systematic review of 62 studies including 46,952,813 patients with cancer, grouped cancer sites into three groups using 5-year relative survival rates: good (5-year survival rates >90%), intermediate (5-year survival rates 50–90%), and poor (5-year survival rates <50%), and reported that the risk of suicide was higher among patients with a poor prognosis.^20^ Using a similar approach to Heinrich et al, studies in Denmark and Taiwan reported that, while both men and women were at high risk for suicide with poor prognosis, women were at high risk even with breast cancer with a good prognosis, which suggested the existence of factors for suicide risk that differ between men and women.^7^^,^^15^

In Japan, previous studies have reported suicide risk among patients with cancer, but few studies have used large-scale data.^21^^–^^23^ More recently, suicide risks were assessed using large-scale data from Japan’s National Cancer Registry, but these studies estimated suicide risk for men and women combined and had a short observation period for each patient.^24^^,^^25^ In addition, although studies of suicide risk using the cancer stage have been conducted in Japan,^24^^,^^25^ to our knowledge, there have been few studies using 5-year relative survival rates for cancer sites. Cancer incidence, mortality, and survival differ by country and sex,^26^^,^^27^ making it significant to examine these rates separately for men and women in Japan.

Therefore, we aimed to investigate the risk of suicide mortality by sex among patients with cancer in Japan using large-scale data from the Osaka Cancer Registry, which was collected for a maximum of 10 years per patient.

## METHODS

### Study design

We conducted a retrospective population-based study using data from the Osaka Cancer Registry (OCR) from January 1, 1985 to December 31, 2013.

### OCR database

Osaka Prefecture is the third largest prefecture in Japan, after Tokyo and Kanagawa, with a population of 8,782,000 and 2,432,000 people aged 65 years and over (aging rate: 27.7%).^28^ The OCR has collected data on the incidence of cancer from all medical institutions in Osaka Prefecture since December 1962. Data from cancer death certificates were obtained through the Department of Public Health and Medical Affairs, Osaka Prefectural Government. Through this process, the OCR collects data on the date of death. The OCR has progressively undertaken follow-ups in 5- and 10-year surveys to determine vital status from registration with the cooperation of local governments. The Act on the Promotion of Cancer Registries was enacted in Japan on December 6, 2013. The law was enforced in 2016 and requires all hospitals in Japan to submit primary data of newly encountered patients with cancer to the National Cancer Registry. Since 2016, the OCR has become part of the National Cancer Registry and has been operating until now.

### The Neoplasms and other causes of Death (NANDE) database

The OCR database for this study lacked data on causes of death. Therefore, we created the NANDE database^29^^–^^31^ by merging the OCR data with the causes of death from Vital Statistics—maintained by the Japanese Ministry of Health, Labour, and Welfare—to cover all causes of death. In the OCR, cancer diagnoses were coded according to the International Classification of Diseases for Oncology, 3rd Edition (ICD-O-3). We converted ICD-O-3 codes to ICD-10 codes using the International Association of Cancer Registries CanReg Tools.^32^ The causes of death were recorded in the Vital Statistics of Japan based on ICD-9 codes between 1985 and 1994, and ICD-10 codes since 1995. The NANDE database was linked by matching sex, date of birth, date of death, and prefecture code and city codes at the time of death. Data from the OCR and Vital Statistics were processed independently in accordance with the Act on Promotion of Cancer Registries and in accordance with the Statistics Act, respectively. All data were de-identified.

### Study population

We obtained data on patients with cancer in the Osaka Prefecture from 1985 to 2016 but only used data through 2013, which was the year in which the follow-ups were completed. Patients were excluded if they had the following: in-situ carcinoma (ICD-10 codes D00–D48), unknown date of death, unknown last date of vital status, unknown date of diagnosis, age at diagnosis not being 15–94 years or unknown age at diagnosis, death certificate notification or death certificate only, unknown survival time, a survival time of 0, and multiple primaries (simultaneous or synchronous; simultaneous means that a second tumor is diagnosed on the same day [0 days] of the diagnosis of the first tumor; synchronous means that a second tumor is diagnosed within 2 months [1–60 days] of the diagnosis of the first tumor).

### Follow-up and outcome

Follow-ups started on the date of diagnosis of the first cancer and ended on the date of death, the date of second cancer diagnosis, the date of move out, the date of 10 years, or December 31, 2013, whichever occurred first. The outcome was defined as suicide death, classified by ICD-9 codes (E950 to E959: Suicide and self-inflicted injury) for deaths between 1985 and 1994, and classified by ICD-10 codes (X60 to X84: Intentional self-harm) for deaths between 1995 and 2013. The definition of suicide was not uniform across previous studies, depending on the country in which the study was conducted. In previous studies from the United States^10^^,^^18^^,^^19^ and Japan,^24^^,^^25^ suicide was defined as Suicide and self-inflicted injury (ICD-9, E950–E959) or Intentional self-harm (ICD-10, X60–X84, Y87.0). In contrast, in previous studies from the United Kingdom,^11^^,^^16^ the definition of suicide included Injury undetermined whether accidentally or purposely inflicted (ICD-9, E980–E989) and Event of undetermined intent (ICD-10, Y10–Y34, excluding Y33.9) in addition to the definitions above. For this study, we followed the definition of suicide used in previous Japanese studies.^24^^,^^25^

### Categorical variables

The years of diagnosis were divided into two groups: 1985–1997 and 1998–2013. The reason for separating the years of diagnosis into two groups was that the suicide rate in the general Japanese male population increased dramatically from 1997 to 1998 and remained high thereafter^3^^,^^4^ (eFigure 1). Adopting the division used in a previous study that used data from the Japanese National Cancer Registry, age at diagnosis (in years) was divided into six groups: 15–39, 40–49, 50–59, 60–69, 70–79, and 80–94 years.^25^ Cancer sites were classified into 22 sites according to the ICD-10 and the Cancer Incidence of Japan (2019; Basic Classification Table A).^33^ Mesothelioma,^16^^,^^19^^,^^20^ testis^18^^,^^20^^,^^34^ and bone and soft tissue^19^^,^^35^^,^^36^ were also added, which were reported to have high risk of suicide in previous studies. Referring to previous studies^7^^,^^9^^,^^15^ the cancer prognosis of these 25 sites was further classified into three groups according to 5-year relative survival rates: good (>70%), moderate (40–70%), and poor (<40%). In contrast, previous studies had the following classifications with 5-year relative survival rates: good (>66%), moderate (33–66%), and poor (<33%).^7^^,^^9^^,^^15^ The 5-year relative survival rates were based on data from the National Cancer Center Cancer Information Service Regional Cancer Registries^37^ and the cancer survival rates at the Japanese Association of Clinical Cancer Centers.^38^ Cancer sites other than the 25 sites were grouped into “other” (Table 1). The stage at diagnosis was divided into six groups: localized, regional lymph node, regional extension, distant, unknown, and unstaged. The unstaged condition was applied to leukemia or multiple myeloma.

**Table 1.** Cancer sites grouped according to the cancer prognosis by cancer site measured by good (>70%), moderate (40–70%), poor (<40%), and other 5-year relative survival in Japan for cancers diagnosed from 1993–2011

Prognosis groups (5-year relative survival rate)	Cancer sites (ICD-10 code)
Good (>70%)	Larynx (C32), bone and soft tissue (C40–C41, C49), skin (C43–C44), breast (C50), cervix uteri (C53), corpus uteri (C54), prostate (C61), testis (C62), bladder^a^ (C67), thyroid (C73)
Moderate (40–70%)	Lip, oral cavity, and pharynx (C00–C14); stomach (C16); colon and rectum (C18–C20); ovary (C56); kidney and urinary tract (C64–C66, C68); malignant lymphoma (C81–C85, C96)
Poor (<40%)	Esophagus (C15), liver and intrahepatic bile ducts (C22), gallbladder and other biliary tract (C23–C24), pancreas (C25), lung and bronchus (C33–C34), brain and other parts of central nervous system (C70–C72), multiple myeloma (C88, C90), leukemia (C91–C95), mesothelioma (C45)
Other	Other than above (C17, C21, C26, C30, C31, C37–C39, C46–C48, C51–C52, C55, C57–C58, C60, C63, C69, C74–C80, C86)

^a^The average 5-year relative survival rate of Japanese women with bladder cancer from 1993 to 2011 was 67.1%. However, we also classified these patients into the good prognosis group along with men with bladder cancer since the average 5-year relative survival rate of male bladder cancer from 1993 to 2011 was 78.4%.

### Statistical methods

Standardized mortality ratios (SMRs) were calculated as the ratio of observed suicides among patients with cancer to the number of expected suicides in the general population.^39^^,^^40^ The 95% confidence intervals (CIs) were calculated by assuming that the observed number of suicides followed a Poisson distribution. The number of expected deaths was calculated by applying the national suicide mortality rates stratified by sex, age, and calendar year. Age was grouped by 5-year periods, and calendar year consisted of 1 year. The suicide rate in the Japanese general population were computed using the World Health Organization’s mortality database.^41^ SMRs were calculated by total, sex, years of diagnosis, age at diagnosis, stage at diagnosis, cancer prognosis groups, and site of cancer. Mortality rate ratios (RRs) and 95% CIs were estimated using Poisson regression analysis modeling with person-years as the model offset. Poisson regression analyses were performed using the number of suicide deaths as the dependent variable and categorical variables (sex, years of diagnosis, age at diagnosis, stage at diagnosis, and cancer prognosis groups) as independent variables. Analyses were adjusted for sex, years of diagnosis, age at diagnosis, cancer prognosis groups, and stage at diagnosis. All analyses were performed using Stata (version 17.0; Stata Corp, College Station, TX, USA).

## RESULTS

**Figure 1.**
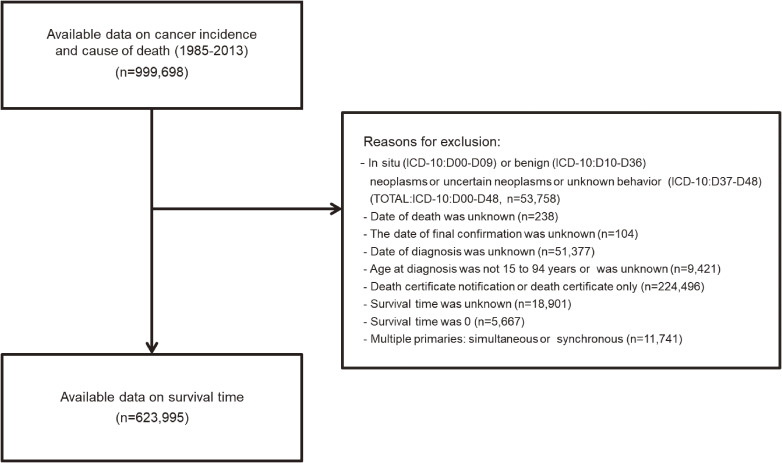
Flowchart of the study population. ICD-10, International Classification of Diseases, 10^th^ revision.

In total, 999,698 patients had a year of diagnosis between 1985 and 2013. Of these, 375,703 patients who met the exclusion criteria were excluded. Consequently, the final analysis included 623,995 patients (Figure 1).

Table 2 shows the baseline characteristics of the study population. Of the 623,995 patients with cancer, 56.8% were male and 43.2% were female. The years of diagnosis were from 1998 to 2013 for more than 70% of both men and women. Age at diagnosis was 60 years or older in 75.4% of men and 63.3% of women. Localized was the most common stage at diagnosis for both men and women (41.2% of men and 43.5% of women), followed by distant for men (20.4%) and regional lymph node for women (15.9%).

**Table 2.** Characteristics of patients with cancer from 1985–2013 in Osaka, Japan

Characteristic	Total		Men		Women	

	*n*	%	*n*	%	*n*	%
All patients	623,995	100	354,126	100	269,869	100
Years of diagnosis						
1985–1997	179,060	28.7	102,179	28.9	76,881	28.5
1998–2013	444,935	71.3	251,947	71.1	192,988	71.5
Age at diagnosis, years						
15–39	23,530	3.8	8,280	2.3	15,250	5.7
40–49	51,846	8.3	18,913	5.3	32,933	12.2
50–59	110,695	17.7	60,176	17.0	50,519	18.7
60–69	186,734	29.9	117,805	33.3	68,929	25.5
70–79	175,749	28.2	109,786	31.0	65,963	24.4
80–94	75,441	12.1	39,166	11.1	36,275	13.4
Stage at diagnosis						
Localized	263,559	42.2	146,050	41.2	117,509	43.5
Regional lymph nodes	85,472	13.7	42,448	12.0	43,024	15.9
Regional extension	89,106	14.3	52,419	14.8	36,687	13.6
Distant	114,724	18.4	72,113	20.4	42,611	15.8
Unknown	59,350	9.5	34,380	9.7	24,970	9.3
Unstaged	11,784	1.9	6,716	1.9	5,068	1.9

Table 3 shows the SMRs and RRs for suicide according to sex, years of diagnosis, and age at diagnosis. During 2,349,432 person-years of follow-up, 1,210 patients with cancer (867 men and 343 women) died by suicide. The overall crude suicide rate was 51.5 per 100,000 person-years (72.5 and 29.7 for men and women, respectively). The overall SMR was 1.66 (95% CI, 1.57–1.75). The SMR for men was 1.66 (95% CI, 1.55–1.77) and 1.65 (95% CI, 1.48–1.83) for women. Contrastingly, the RR for men (vs women) was 2.15 (95% CI, 1.89–2.45).

**Table 3.** Observed numbers of suicide deaths and standardized mortality ratio for patients with cancer by years of diagnosis and age at diagnosis

Characteristic		Person years	Suicide death				SMR	95% CI		RR^c^	95% CI	

			Patients with cancer		General population							
		
			Obs	Rate^a^	Exp	Rate^b^		Lower	Upper		Lower	Upper
Men	Years of diagnosis											
	1985–1997	381,354	313	82.1	165.1	43.3	1.90	1.70	2.12	1.00	Reference	
	1998–2013	814,788	554	68.0	357.3	43.8	1.55	1.43	1.69	0.83	0.72	0.95
	Age at diagnosis, years											
	15–39	42,901	23	53.6	13.0	30.3	1.77	1.18	2.66	1.00	Reference	
	40–49	86,943	70	80.5	39.6	45.6	1.77	1.40	2.23	1.40	0.87	2.25
	50–59	250,628	203	81.0	126.0	50.3	1.61	1.40	1.85	1.40	0.90	2.16
	60–69	418,461	324	77.4	172.9	41.3	1.87	1.68	2.09	1.36	0.89	2.09
	70–79	320,220	188	58.7	129.6	40.5	1.45	1.26	1.67	1.08	0.70	1.67
	80–94	76,989	59	76.6	41.3	53.7	1.43	1.11	1.84	1.44	0.88	2.34
Women	Years of diagnosis											
	1985–1997	390,374	122	31.3	78.0	20.0	1.56	1.31	1.87	1.00	Reference	
	1998–2013	762,916	221	29.0	130.2	17.1	1.70	1.49	1.94	0.88	0.70	1.10
	Age at diagnosis, years											
	15–39	89,074	16	18.0	10.3	11.5	1.56	0.95	2.54	1.00	Reference	
	40–49	190,606	43	22.6	26.5	13.9	1.62	1.20	2.19	1.27	0.71	2.25
	50–59	265,423	93	35.0	43.4	16.3	2.15	1.75	2.63	1.88	1.10	3.20
	60–69	298,266	106	35.5	55.3	18.5	1.92	1.59	2.32	1.85	1.09	3.15
	70–79	228,850	62	27.1	51.7	22.6	1.20	0.93	1.54	1.40	0.80	2.44
	80–94	81,072	23	28.4	21.2	26.1	1.09	0.72	1.63	1.47	0.77	2.81
Sex	Men	1,196,142	867	72.5	522.4	43.7	1.66	1.55	1.77	2.15	1.89	2.45
	Women	1,153,290	343	29.7	208.3	18.1	1.65	1.48	1.83	1.00	Reference	
Total		2,349,432	1,210	51.5	730.7	31.1	1.66	1.57	1.75	—	—	—

CI, confidence interval; Exp, expected deaths; Obs, observed deaths; RR, rate ratio; SMR, standardized mortality ratio.

^a^Mortality rate of patients with cancer per 100,000 person years = Obs / person years * 100,000.

^b^Mortality rate of general population per 100,000 person years = Exp / person years * 100,000.

^c^Multivariate models included the factors years of diagnosis, age at diagnosis, cancer stage, and prognosis of cancer site measured by 5-year relative survival.

Table 4 shows the SMRs and RRs for suicide in men according to the cancer prognosis groups, site, and stage at diagnosis. The SMRs for cancer prognosis groups in men were 1.01 (95% CI, 0.84–1.22) in the good group, 1.53 (95% CI, 1.39–1.68) in the moderate group, and 2.54 (95% CI, 2.27–2.85) in the poor group. By cancer site, the SMRs of all cancer sites in the good prognosis group did not increase significantly. The SMRs in the moderate prognosis group were significantly increased, except in the kidneys and urinary tract. The SMRs in the poor prognosis group, except for leukemia, were significantly increased in the following order: mesothelioma, pancreas, esophagus, gallbladder and other biliary tract, lung and bronchus, brain and other parts of the central nervous system, multiple myeloma, and liver and intrahepatic bile ducts. By stage at diagnosis, SMRs significantly increased with advanced-stage cancer. Similarly, the RRs were significantly increased in the order of worse prognosis and advanced-stage cancer.

**Table 4.** Observed numbers of suicide deaths and standardized mortality ratio for male patients with cancer by cancer prognosis groups, site, and stage at diagnosis

Characteristic	*n*	Person years	Suicide death				SMR	95% CI		RR^c^	95% CI	

			Patients with cancer		General population							
		
			Obs	Rate^a^	Exp	Rate^b^		Lower	Upper		Lower	Upper
Prognosis groups; Site (first primary cancer)												
Good	57,149	257,759	111	43.1	109.7	42.6	1.01	0.84	1.22	1.00	Reference	
Larynx	4,511	22,894	8	34.9	10.1	44.3	0.79	0.39	1.58			
Bone and soft tissue	1,372	5,840	2	34.2	2.2	38.0	0.90	0.23	3.60			
Skin	3,752	16,911	10	59.1	7.5	44.1	1.34	0.72	2.49			
Breast	291	1,505	0	0.0	0.7	43.2	0.00	N/A	N/A			
Prostate	31,918	133,507	63	47.2	56.0	41.9	1.13	0.88	1.44			
Testis	1,740	10,931	4	36.6	3.9	35.3	1.04	0.39	2.76			
Bladder	11,466	55,250	20	36.2	24.7	44.8	0.81	0.52	1.25			
Thyroid	2,099	10,921	4	36.6	4.7	42.8	0.86	0.32	2.28			
Moderate	158,915	648,958	438	67.5	286.9	44.2	1.53	1.39	1.68	1.42	1.15	1.76
Lip, oral cavity, and pharynx	9,399	35,270	45	127.6	15.7	44.4	2.87	2.14	3.85			
Stomach	77,714	310,651	208	67.0	137.6	44.3	1.51	1.32	1.73			
Colon and rectum	53,223	233,648	151	64.6	103.6	44.3	1.46	1.24	1.71			
Kidney and urinary tract	9,210	37,617	13	34.6	16.6	44.2	0.78	0.45	1.35			
Malignant lymphoma	9,369	31,772	21	66.1	13.5	42.4	1.56	1.02	2.39			
Poor	131,202	269,724	298	110.5	117.3	43.5	2.54	2.27	2.85	2.25	1.81	2.81
Esophagus	13,692	29,908	49	163.8	13.4	44.9	3.65	2.76	4.83			
Liver and intrahepatic bile ducts	35,415	86,900	61	70.2	37.7	43.3	1.62	1.26	2.08			
Gallbladder and other biliary tract	6,465	11,567	15	129.7	5.1	43.8	2.96	1.78	4.91			
Pancreas	10,869	11,041	18	163.0	4.8	43.7	3.73	2.35	5.92			
Lung and bronchus	55,190	107,168	133	124.1	47.0	43.8	2.83	2.39	3.36			
Brain and other parts of the central nervous system	1,816	4,844	5	103.2	1.8	37.8	2.73	1.14	6.56			
Multiple myeloma	1,763	4,823	5	103.7	2.1	43.3	2.39	1.00	5.74			
Leukemia	5,013	12,229	6	49.1	4.9	40.1	1.23	0.55	2.73			
Mesothelioma	979	1,245	6	481.9	0.6	45.0	10.72	4.82	23.86			
Other	6,860	19,701	20	101.5	8.5	43.1	2.35	1.52	3.65	2.01	1.25	3.25

Stage at diagnosis												
Localized	146,050	710,079	380	53.5	310.2	43.7	1.23	1.11	1.35	1.00	Reference	
Regional lymph nodes	42,448	156,436	145	92.7	69.0	44.1	2.10	1.79	2.47	1.62	1.33	1.96
Regional extension	52,419	121,234	136	112.2	52.4	43.2	2.59	2.19	3.07	1.96	1.61	2.40
Distant	72,113	89,645	125	139.4	38.9	43.4	3.21	2.70	3.83	2.45	1.99	3.00
Unknown	34,380	101,964	70	68.7	45.1	44.2	1.55	1.23	1.96	1.24	0.96	1.60
Unstaged	6,716	16,784	11	65.5	6.9	40.9	1.60	0.89	2.89	0.83	0.45	1.54

CI, confidence interval; Exp, expected deaths; N/A, not applicable; Obs, observed deaths; RR, rate ratio; SMR, standardized mortality ratio.

^a^Mortality rate of patients with cancer per 100,000 person years = Obs / person years * 100,000.

^b^Mortality rate of general population per 100,000 person years = Exp / person years * 100,000.

^c^Multivariate models included the factors years of diagnosis, age at diagnosis, cancer stage, and prognosis groups of cancer site measured by 5-year relative survival.

**Figure 2.**
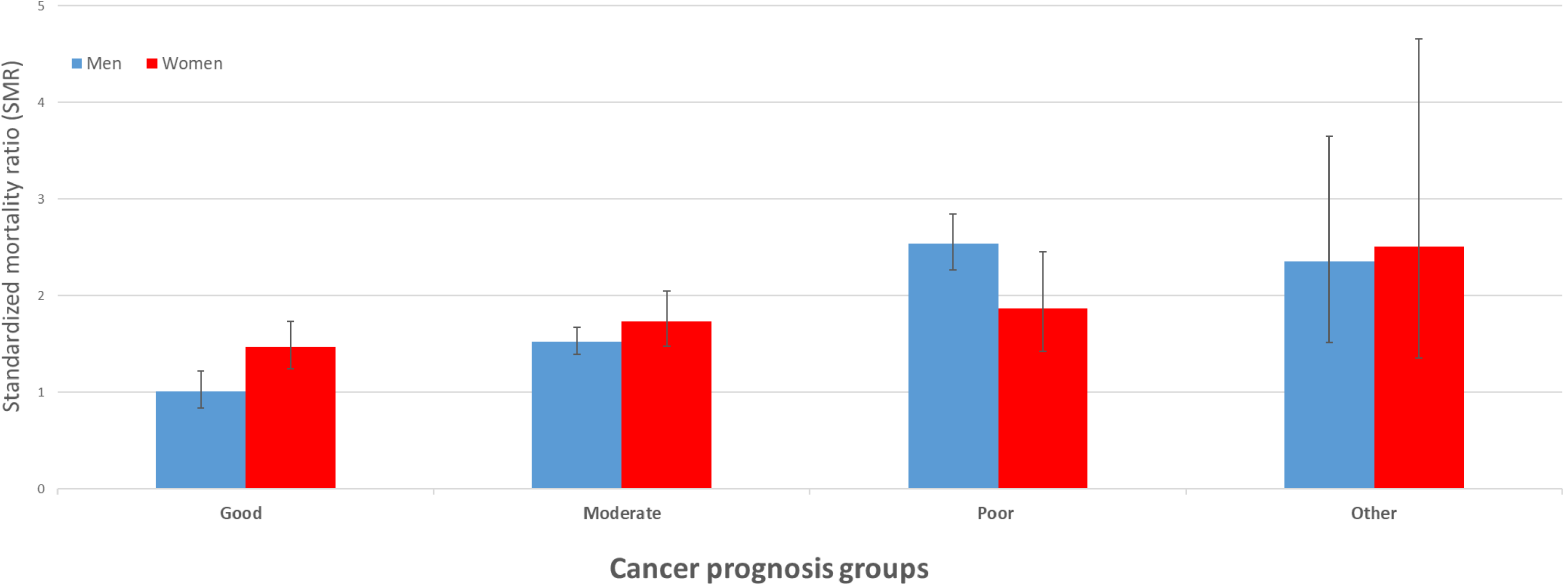
Standardized mortality ratio for cancer prognosis groups by sex. SMR, standardized mortality ratio.

**Figure 3.**
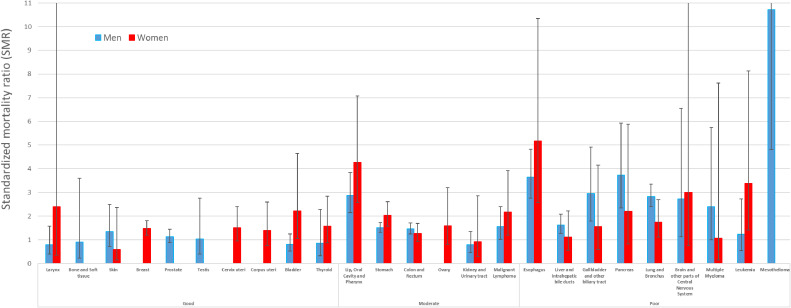
Standardized mortality ratio for cancer site by sex. SMR, standardized mortality ratio.

**Figure 4.**
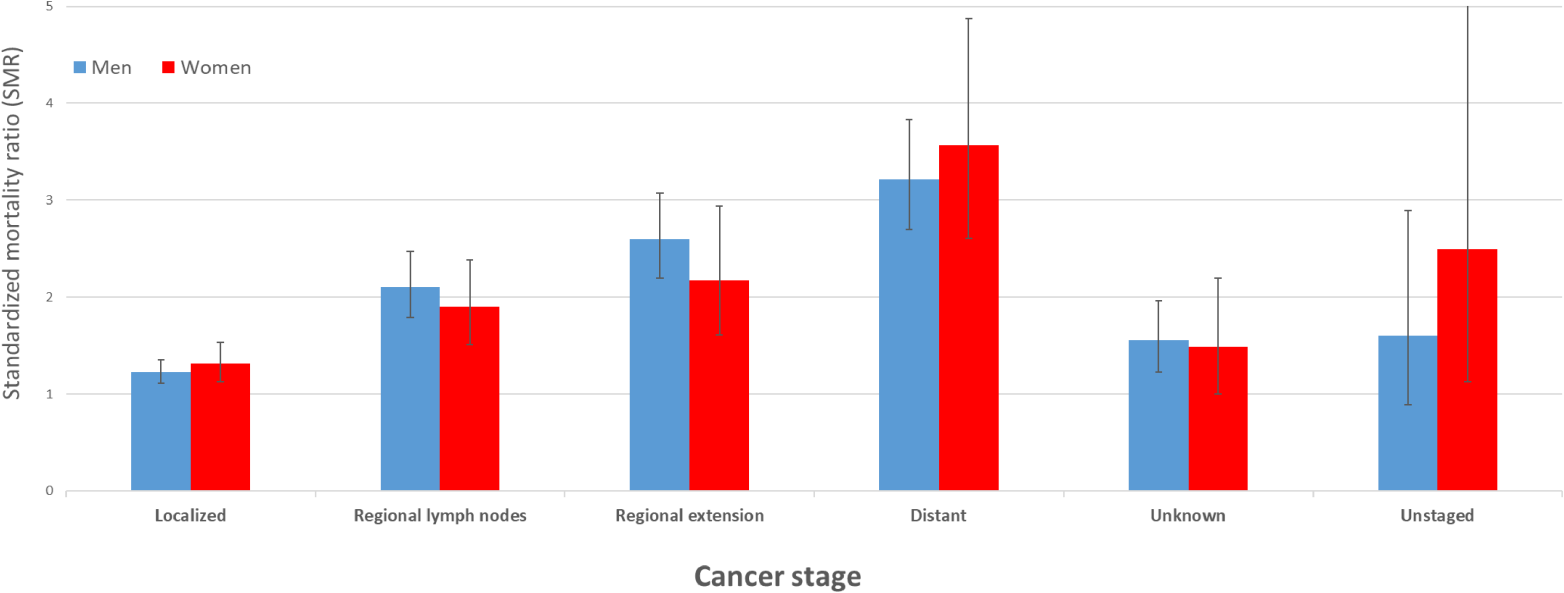
Standardized mortality ratio for cancer stage by sex. SMR, standardized mortality ratio.

Table 5 shows the SMRs and RRs for suicide in women according to the cancer prognosis groups, site, and stage at diagnosis. Concerning cancer prognosis groups, (1) the SMRs for women were 1.47 (95% CI, 1.24–1.73) in the good group, 1.74 (95% CI, 1.47–2.05) in the moderate group, and 1.87 (95% CI, 1.43–2.46) in the poor group, and (2) the SMRs in the good prognosis group for women were significantly increased in the following order: bladder and breast. The SMRs in the moderate prognosis group were significantly increased in the following order: lip, oral cavity, and pharynx; malignant lymphoma; and stomach. The SMRs in the poor prognosis group were significantly increased in the following order: esophagus, leukemia, lung, and bronchus. By stage at diagnosis, SMRs were significantly increased in female patients with advanced-stage cancer as much as in men. However, the trend in the RRs for cancer prognosis groups differed between men and women. The RRs of all prognosis groups for women did not increase significantly. In contrast, the RRs of the stage at diagnosis were significantly increased in women as well as in men with advanced-stage cancer. See also Figure 2, Figure 3, and Figure 4 for a visualization of the results in Table 4 and Table 5.

**Table 5.** Observed numbers of suicide deaths and standardized mortality ratio for female patients with cancer by cancer prognosis groups, site, and stage at diagnosis

Characteristic	*n*	Person years	Suicide death				SMR	95% CI		RR^c^	95% CI	

			Patients with cancer		General population							
		
			Obs	Rate^a^	Exp	Rate^b^		Lower	Upper		Lower	Upper
Prognosis groups; Site (first primary cancer)												
Good	97,759	556,000	138	24.8	94.1	16.9	1.47	1.24	1.73	1.00	Reference	
Larynx	373	2,000	1	50.0	0.4	21.0	2.39	0.34	17.00			
Bone and soft tissue	1,082	4,924	0	0.0	0.8	15.8	0.00	N/A	N/A			
Skin	3,687	16,938	2	11.8	3.4	20.0	0.59	0.15	2.36			
Breast	60,874	362,042	89	24.6	60.4	16.7	1.47	1.20	1.81			
Cervix uteri	12,859	70,414	18	25.6	11.9	16.9	1.51	0.95	2.40			
Corpus uteri	8,592	42,854	10	23.3	7.2	16.7	1.40	0.75	2.60			
Bladder	3,301	14,819	7	47.2	3.2	21.3	2.22	1.06	4.65			
Thyroid	6,991	42,010	11	26.2	7.0	16.6	1.58	0.87	2.85			
Moderate	101,694	430,889	143	33.2	82.3	19.1	1.74	1.47	2.05	1.22	0.95	1.55
Lip, oral cavity, and pharynx	4,169	18,881	15	79.4	3.5	18.6	4.26	2.57	7.07			
Stomach	37,609	154,653	62	40.1	30.5	19.7	2.03	1.58	2.61			
Colon and rectum	40,582	180,034	44	24.4	35.0	19.4	1.26	0.94	1.69			
Ovary	7,579	31,243	8	25.6	5.0	16.0	1.60	0.80	3.20			
Kidney and urinary tract	4,192	17,204	3	17.4	3.3	18.9	0.92	0.30	2.86			
Malignant lymphoma	7,563	28,875	11	38.1	5.1	17.5	2.17	1.20	3.92			
Poor	63,632	144,865	52	35.9	27.8	19.2	1.87	1.43	2.46	1.25	0.88	1.77
Esophagus	3,021	7,882	8	101.5	1.6	19.7	5.17	2.59	10.34			
Liver and intrahepatic bile ducts	14,226	36,409	8	22.0	7.2	19.7	1.11	0.56	2.22			
Gallbladder and other biliary tract	7,170	12,090	4	33.1	2.6	21.2	1.56	0.59	4.16			
Pancreas	8,858	9,298	4	43.0	1.8	19.5	2.21	0.83	5.88			
Lung and bronchus	23,470	60,121	20	33.3	11.5	19.1	1.74	1.12	2.69			
Brain and other parts of the central nervous system	1,506	4,323	2	46.3	0.7	15.5	3.01	0.75	12.02			
Multiple myeloma	1,582	4,879	1	20.5	0.9	19.1	1.07	0.15	7.62			
Leukemia	3,487	9,375	5	53.3	1.5	15.8	3.38	1.41	8.12			
Mesothelioma	312	487	0	0.0	0.1	16.4	0.00	N/A	N/A			
Other	6,784	21,536	10	46.4	4.0	18.5	2.51	1.35	4.66	1.66	0.87	3.19

Stage at diagnosis												
Localized	117,509	663,592	157	23.7	119.8	18.0	1.31	1.12	1.53	1.00	Reference	
Regional lymph nodes	43,024	219,307	74	33.7	39.0	17.8	1.90	1.51	2.38	1.44	1.09	1.90
Regional extension	36,687	105,161	42	39.9	19.3	18.4	2.18	1.61	2.94	1.62	1.15	2.28
Distant	42,611	60,147	39	64.8	11.0	18.2	3.56	2.60	4.88	2.55	1.78	3.64
Unknown	24,970	90,850	25	27.5	16.8	18.5	1.49	1.00	2.20	1.15	0.75	1.75
Unstaged	5,068	14,233	6	42.2	2.4	16.9	2.49	1.12	5.55	1.67	0.70	3.94

CI, confidence interval; Exp, expected deaths; N/A, not applicable; Obs, observed deaths; RR, rate ratio; SMR, standardized mortality ratio.

^a^Mortality rate of patients with cancer per 100,000 person years = Obs / person years * 100,000.

^b^Mortality rate of general population per 100,000 person years = Exp / person years * 100,000.

^c^Multivariate models included the factors years of diagnosis, age at diagnosis, cancer stage, and prognosis groups of cancer site measured by 5-year relative survival.

## DISCUSSION

In our population-based study of 623,995 patients with cancer from 1985 to 2013, we found that patients with cancer had an equally high risk of suicide for both men and women compared to the general population. Contrastingly, among patients with cancer, men had more than twice the risk of suicide as women. The SMRs were higher in advanced-stage cancer as compared to the general population for both sexes. In terms of SMR for cancer prognosis groups, men with a good prognosis had little difference in risk from the general population, while men with a poor prognosis had a high SMR. In contrast, women had a smaller difference in risk in the prognosis groups than men. Therefore, the results of this study suggest that there are differences in the SMR patterns for cancer prognosis groups by sex.

The results of the SMRs for men and women in the previous studies were divided into three groups. First, the SMR for men is higher than that for women.^7^^,^^9^^–^^11^^,^^14^^,^^15^^,^^17^ Second, the SMRs for men and women are comparable,^6^^,^^12^^,^^13^^,^^16^^,^^19^^,^^21^^,^^24^^,^^25^ which is consistent with the current results. Third, the SMR in women is higher than that in men.^18^ However, we should be careful about directly comparing the SMRs of other studies; the SMR values are influenced by observation time or suicide rates in the general population and patients with cancer. Contrastingly, in a risk comparison among patients with cancer, previous overseas studies^12^^,^^16^^,^^18^ reported that the suicide risk for men was higher than that for women, which is consistent with the current results. However, a previous Japanese study reported that the RR for women (vs men) was only 1.04 (95% CI, 0.85–1.29), which was inconsistent with the current results.^25^ This may be owing to the differences in the time of observation. The difference in suicide rates between men and women in the general population during the observation period of this study (eFigure 1) was larger than that of the previous study from 2016 to 2018,^5^ which may be one reason for the larger difference in suicide rates between men and women in patients with cancer in this study. In Japan, the evidence on suicide among patients with cancer by sex is scant, and further study is needed.

The results for the cancer stage and prognosis groups in this study were consistent with previous studies.^7^^,^^15^ To our knowledge, the underlying mechanism by which cancer diagnosis increases the risk of suicide is unknown. However, previous studies of suicidal ideation in patients with advanced-stage cancer have reported associations with depressive disorder^42^ and pain.^42^^,^^43^ Therefore, increased patient symptoms (pain, depression) may increase suicide risk regardless of sex and anatomical site, which may explain the higher SMRs for advanced-stage cancer, or poor prognosis sites in this study. Furthermore, head and neck cancers, including those of the oral cavity and pharynx, quickly manifest themselves on the external surface, which may reduce patients’ quality of life and increase the risk of suicide, regardless of sex.^44^

Contrastingly, suicide risk for female patients with breast and bladder cancers, which have a good prognosis, may be the result of a women-specific mechanism different from those described above. The loss or absence of a breast caused by cancer treatment is thought to be a source of stress specific to women. A British systematic review of the evidence on adverse mental health outcomes in breast cancer survivors (>1 year) reported that female patients with breast cancer were at increased risk for anxiety, depression, suicide, and neurocognitive and sexual dysfunction as compared to women without cancer.^45^ A retrospective study using the Surveillance Epidemiology and End Results database in the United States reported that surgery was an independent risk factor for suicide among American patients with breast cancer.^46^ A national cohort study using data from the Health Insurance Review and Assessment Service in Korea reported that South Korean patients undergoing mastectomy for breast cancer experienced depression more frequently than healthy individuals.^47^ Unfortunately, we did not discuss data on surgery in our study. Mastectomy may have a negative impact on body image among female patients with breast cancer and may increase the risk of suicide.

Regarding female patients with bladder cancer, the 5-year relative survival rate, which was lower than that of men, may have affected the suicide risk. The average 5-year relative survival rate for female patients with bladder cancer in Japan from 1993 to 2011 was 67.1%—more than 10% lower than that for men (78.4%).^37^ A study comparing Japan with the United States and Europe reported that the 5-year relative survival rate of female patients with bladder cancer decreased after the age of 65 years in Japan.^48^ All seven suicides among female patients with bladder cancer in this study were age 65 or older at death, suggesting that two prognostic factors, “female” and “older age,” may have influenced the increased risk of suicide. In this study, we used a common method for men and women to classify cancer sites into prognosis groups, which may not have been an optimal classification method because some sites have different 5-year relative survival rates among both sexes.

Suicide rates in Japan are more responsive to economic factors,^49^ resulting in male suicide rates being higher than female suicide rates. However, the female suicide rate in Japan is among the highest in OECD countries.^2^ Therefore, suicide prevention strategies specific to women and not only to men are needed. By contributing to screening for suicide among both men and women, our study provides a basis for clinical and public health strategies aimed at suicide prevention among male and female patients with cancer in Japan. On this basis, both men and women should be aware of suicide screening for advanced or poor prognosis cancers, but women should also be aware of sites that are not associated with cancer prognosis. For example, appearance care for patients with breast cancer improves anxiety and depression and enhances well-being.^50^^,^^51^ Persons involved in suicide prevention should assess the psychological distress during the breast cancer treatment process and consider incorporating appearance care. In addition, suicide is a sensitive issue that is more difficult to discuss in clinical settings than screening for depression, pain, or anxiety. In Japan, suicide has not historically been criminalized. Therefore, there are no legal restrictions on having direct conversations with patients about suicide. In contrast, suicide has historically been a crime or stigma in some countries, which requires consideration and response in accordance with national culture and norms.

Our study had some limitations. First, we did not adjust for the potential risk factors for suicide (psychiatric illness,^52^ history of tobacco use,^53^ alcohol consumption history,^54^ unemployment,^55^ family history of suicide,^56^ living alone,^57^ and bereavement^58^). These risk factors may have affected the results of this study. Second, we have not examined the data on cancer treatment, pre-existing conditions, or comorbidities. Previous studies reported that there is limited evidence concerning the impact of pre-existing psychiatric conditions on suicide risk among patients with cancer.^59^^–^^61^ Also, a Canadian population-based, retrospective matched cohort study reported that patients with cancer had an increased risk of suicide, even if they had visited a psychiatric outpatient clinic prior to diagnosis.^62^ Third, it is difficult to distinguish between accidental death and suicide, which may lead to the misclassification of the cause of death. A study comparing the annual proportional change of suicide, undetermined death, and accidental death in South Korea with Japan and Hong Kong reported that 43% of the increase in South Korean suicides was associated with the misclassification of accidental deaths but that Japan had fewer misclassifications than South Korea.^63^ Fourth, the generalizability of the results of this study is limited. This study was based on data from Osaka Prefecture over 10 years ago, so caution should be applied when extending these findings to current context in the Osaka Prefecture or other regions. Fifth, we did not retain data on cancer notification in this study. In Japan, cancer notification rates were less than 20% (before 1993), 28.6% (around 1995), and 73.5% (around 2012).^64^ The results of this study may change depending on whether or not cancer was notified to patients. Sixth, the analysis population was about 60% of all patients, with 40% excluded (Figure 1). Therefore, the distribution of suicides among the excluded patients and their survival times may change the results of this study. However, before exclusion, suicide accounted for 0.30% of all deaths (2,022 suicides/678,323 deaths), which was almost equal to 0.32% of deaths analyzed in this study (1,210 suicides/376,281 deaths), suggesting that the impact of the excluded patients on the results may not be significant. Seventh, the basis for classifying cancer sites for the cancer prognosis groups in this study was not the annual 5-year relative survival rates. However, the 5-year relative survival data in this study did not significantly change the ranking of prognoses.^37^ Therefore, we would expect the differences in the results of the cancer prognosis groups in this study to be small even when using the annual 5-year relative survival rates.

### Conclusions

In this population, the SMRs by sex were almost equal, and both sexes with advanced cancer were at a higher risk of suicide; for the SMRs by cancer prognosis, both sexes with a poor prognosis had a higher suicide risk, but the difference in SMRs between the good and poor prognosis groups was smaller for women than men.

## SUPPLEMENTARY MATERIAL

eFigure 1. Age-standardized suicide mortality rates for the general population in Japan, 1985–2013.
