# Supplementary figures and images for: Suicide Risk Among Patients With Cancer by Sex in Japan: A Population-based Study
Source: J Epidemiol. 2024 Nov 5;34(11):505–14. doi: 10.2188/jea.JE20230280 (PMC11464848; doi:10.2188/jea.JE20230280)

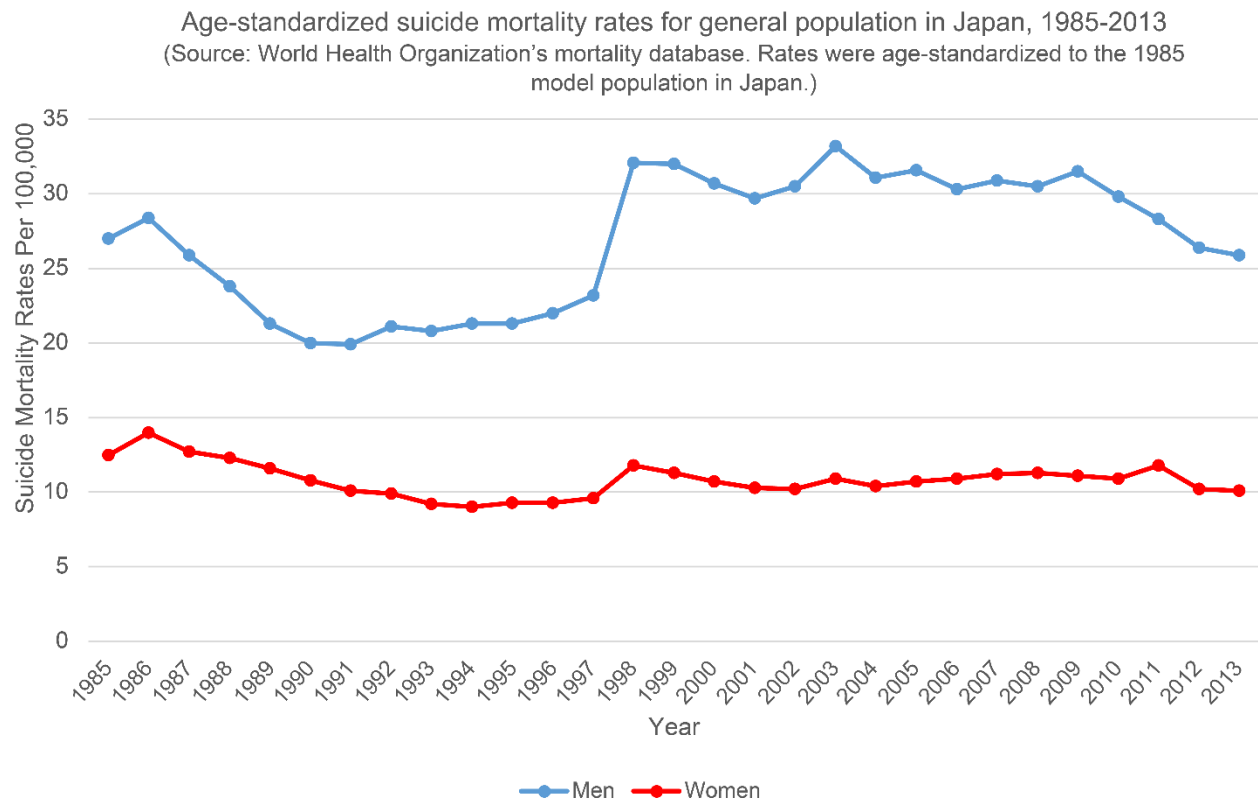

**eFigure 1.** Age-standardized suicide mortality rates for the general population in Japan, 1985–2013

Supplement: Supplementary file 1 [file je-34-505-s001.pdf]
